# Increased Cerebello-Prefrontal Connectivity Predicts Poor Executive Function in Congenital Heart Disease

**DOI:** 10.3390/jcm12165264

**Published:** 2023-08-12

**Authors:** Aurelia Sahel, Rafael Ceschin, Daryaneh Badaly, Madison Lewis, Vince K. Lee, Julia Wallace, Jacqueline Weinberg, Vanessa Schmithorst, Cecilia Lo, Ashok Panigrahy

**Affiliations:** 1 Department of Radiology, University of Pittsburgh, Pittsburgh, PA 15213, USA; aurel117@gmail.com (A.S.); rcc10@pitt.edu (R.C.); mtl47@pitt.edu (M.L.); vkl2@pitt.edu (V.K.L.); julia.wallace@chp.edu (J.W.); vanessa.schmithorst@chp.edu (V.S.); 2 Department of Biomedical Informatics, University of Pittsburgh, Pittsburgh, PA 15206, USA; 3 Child Mind Institute, New York, NY 10022, USA; daryaneh.badaly@childmind.org; 4 Department of Cardiology, University of Pittsburgh, Pittsburgh, PA 15261, USA; jacqueline.weinberg@chp.edu; 5 Department of Developmental Biology, University of Pittsburgh, Pittsburgh, PA 15201, USA; cel36@upmc.edu; 6 Department of Pediatric Radiology, Children’s Hospital of Pittsburgh of UPMC, 45th Street and 4401 Penn Avenue, Pittsburgh, PA 15224, USA

**Keywords:** cerebellum, connectivity, cognitive function, MRI, CHD

## Abstract

Background: Children and adolescents with congenital heart disease (CHD) are at risk for cognitive impairments, such as executive function deficits and motor delays, which can impact their academic and adaptive functioning as well as their quality of life. We investigated whether alterations in connectivity between the prefrontal and cerebellar brain structures exist between CHD and control cohorts and if these alterations could predict cognitive or motor impairment among youths with CHD. Methods: 53 participants with CHD and 73 healthy control participants completed multi-modal magnetic resonance imaging (MRI) of the brain, including high-resolution diffusion tensor imaging at 3T. We measured connectivity from masked regions of interest in the cerebellum to the frontal cortex using a probabilistic tractography method. Participants also completed neuropsychological tests of cognitive and motor skills using the NIH Toolbox. Results: In the CHD group, fractional anisotropy (FA) was increased in the cognitive loop connectivity pathways, including from the right cerebellum to the left thalamus (*p* = 0.0002) and from the left thalamus to the left medial frontal gyrus (MFG) (*p* = 0.0048) compared with the healthy control group. In contrast, there were no differences between CHD and controls in motor loop connectivity pathways. An increase in FA from the right thalamus to the MFG tract in the cognitive loop (posterior subdivision) predicted (*p* = 0.03) lower scores on the NIHTB tests, including those of executive functioning. A transient increase in connectivity of the cognitive loop in the adolescent group was observed relative to the child and adult groups. Conclusions: Our results suggest that selective alteration of cerebellum-cerebral connectivity circuitry within the cognitive loops predicts cognitive dysfunction in CHD youth. Our study suggests a critical period of cerebellar circuitry plasticity in the adolescent period in CHD subjects that drives neurocognitive function. Further replication and validation in other pediatric CHD cohorts is warranted for future work.

## 1. Introduction

The standard of care for patients with congenital heart disease (CHD) has drastically evolved in the past decades, allowing a higher survival rate and longer life expectancy. As such, a new focus on quality of life has emerged, considering developmental and cognitive outcomes from early childhood and increasingly through adulthood. Past studies have demonstrated that, while children with CHD have intellectual abilities within the average range overall, there is increased variability in their intellectual functioning compared with the normative population [1,2,3]. Moreover, children with CHD have higher rates of developmental delays (e.g., with motor and language abilities) and are at greater risk for cognitive concerns (e.g., with visual-spatial skills, pragmatic language skills, and executive functions) [4]. In turn, pediatric CHD populations have difficulties with academic and adaptive skills [5,6,7,8].

Brain dysmaturation secondary to impaired cortical neurogenesis is thought to underlie these developmental and neurocognitive deficits seen in CHD. Multiple studies have shown that, in addition to structural white matter lesions, focal and general brain volume reduction and delayed brain maturation can be seen [9,10,11,12]. When focusing on fetal and neonatal cerebellar brain dysmaturation, volume reduction and anatomical alterations have been demonstrated as early as in the fetus and newborn as well as in pediatric populations [13,14,15,16]. Likewise, CHD preclinical models with impaired cilia motility [17] and human patients also demonstrate impaired subcortical neurogenesis with cerebellar atypicalities [16]. Interestingly, using deep neural network tools, our group set forth that the superior surface of the cerebellum (composed of mostly the posterior lobe and the midline vermis) were regions of vulnerability for brain dysmaturation in CHD patients [18]. These regions are of crucial relevance in relation to the neurodevelopmental outcome of CHD patients [9,10,11,12]. 

Indeed, recent studies have linked cerebral and cerebellar abnormalities with altered neurocognitive outcomes; more specifically, they have shown that lower fractional anisotropy (FA) in the left parietal region, right precentral region, and right frontal region were associated with lower scholastic achievement and abnormal executive functioning in pediatric populations [19]. Likewise, various studies demonstrated that reduced FA in the white matter tracts was associated with impairments in various cognitive domains such as intellectual functioning, processing speed, and memory in adolescents and young adults [20,21]. Furthermore, a connectomic analysis revealed a link between decreased global efficiency and worse executive function and academic achievement [22]. More recently, a study demonstrated that cerebellar volume is highly associated with prefrontal volumes and cognitive measures of executive function, notably working memory [23]. 

Interestingly, while some studies demonstrated a direct link between increased cortical thickness and greater attention control in frontoparietal networks and greater executive function in the cingulo-opercular network [24], others have described that, more specifically, the cerebellum contributes to higher cognitive functions, including executive control and working memory [25]. More specifically, lesions of the posterior lobe of the cerebellum result in the cerebellar cognitive affective syndrome (CCAS), which is typically characterized by deficits in executive function, visual spatial processing, linguistic skills, and regulation of affect [26]. These different cognitive functions have been shown to be impaired in patients with CHD, particularly during the child and adolescent period [4]. 

Here, using a multi-modal neuroimaging approach, we aimed to (1) investigate cerebello-cerebral differences between patients with CHD and healthy, age-matched controls using both morphological and connectivity-based probabilistic mapping of the cognitive and motor loops; and (2) correlate these cerebello-cerebral circuitry measurements with cognitive and motor outcome measures.

## 2. Methods

### 2.1. Participants

A total of 86 healthy controls and 73 patients were recruited for this analysis. Participants were recruited from a single center using print and digital advertisements, an online registry of healthy volunteers, and referrals within targeted clinics. Study exclusion criteria included comorbid genetic disorders, contraindications for MRI (e.g., a pacemaker), and non-English speakers. For healthy controls, study exclusion criteria also included preterm birth and neurological abnormalities (e.g., brain malformations, strokes, and hydrocephalus). Study procedures included magnetic resonance imaging (MRI) of the brain, neuropsychological measures from the NIH Toolbox, and a review of demographic information and medical records. A total of 11 participants with CHD and 10 healthy participants were excluded from the final sample due to refusal to undergo MRI and/or neuropsychological testing, and 7 participants with CHD and 3 healthy controls were excluded due to poor imaging quality (See Scheme S1). Overall, 53% and 35% of the control and CHD cohorts, respectively, are female. Further on, the cohort will be broken into 3 age groups as follows: a school-age child group (age 6–10: n = 19 controls and 10 CHD patients, of which 50% and 30% respectively are female), an adolescent group (age 11–15: n = 32 controls and 16 CHD patients, of which 42% and 35% respectively are female), and a young adult group (age 16 and up: n = 13 controls and 19 CHD patients, of which 57% and 45% are female). All CHD age groups had an approximate 60/40 ratio of cyanotic vs. acyanotic lesion distribution. Participants who had reached the age of majority provided informed consent; minors consented to the project, and their parent or legal guardian provided consent on their behalf. The project was approved by our Institutional Review Board (IRB) and completed in accordance with the ethical principles of the Helsinki Declaration. 

### 2.2. Brain Imaging Procedure

Participants underwent brain MRI on a 3 Tesla Skyra scanner (Siemens, Erlangen, Germany), using a 32-channel head coil. Three-dimensional sagittally acquired 42-direction images (DTI and T1) were used for our analysis. The DTI sequence had the following parameters: FOV = 256 mm, voxel size = 2.0 mm (isotropic), TE/TR = 92 ms/12,600 ms, and 42 directions at B = 1000 s/mm^2^. The T1 sequence had the following parameters: TR = 2400 ms, TE = 3.16 ms, TI = 1200 ms, flip angle = 8°, and 1 mm isotropic voxel size.

### 2.3. Image Processing

We created an automated pipeline for image pre-processing and tractography generation of both motor and cognitive loops emerging from the cerebellum to the cerebral cortex, measured as follows (see Scheme S2).

**Figure 1:**
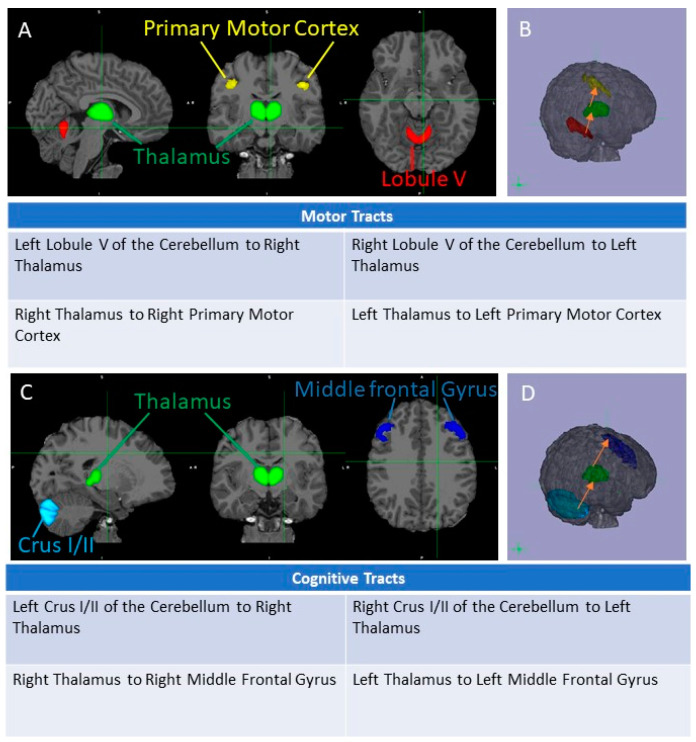
Masks of seed regions to ROI in cognitive and motor loops: Because of contralaterality of projection, left cerebellum projects to right thalamus and vice versa. Motor loop depicted in panel (**A**,**B**). Seed regions denoted as Lobule V of the Cerebellum (in red), the Thalamus (in green), and the primary motor cortex (in yellow). Trajectories (in orange) from seed regions to target regions for each tract in panel (**B**). A total of four tracts per loop were used. Cognitive loop depicted in panel (**C**,**D**). Panel (**C**): Seed regions denoted as CrusI/II of the Cerebellum (in light blue), Thalamus (in green), and Middle frontal gyrus of the Prefrontal Cortex (in dark blue). Trajectories depicted in panel (**D**) as arrows (orange) from seed region to target region.

Masks of the motor part of the cerebellum labeled Lobule V and the cognitively related areas of the cerebellum labeled Crus I and Crus II were generated using automated registration, followed by manual inspection and correction if necessary. Masks of these regions were defined using the SUIT atlas [27,28]. Masks of the thalamus using the tractography-based segmentation of Johansen-Berg and colleagues [29] as well as masks of the left primary motor cortex (PMC) as defined by the Jülich histological atlas [30] and the middle frontal gyrus (MFG) as defined by the Harvard-Oxford cortical atlas [31] were used. All masks’ atlases were available in FSL (Figure 1).

Diffusion-weighted images were processed using the Oxford Centre for Functional Magnetic Resonance Imaging of the Brain (FMRIB) Software Library’s (FSL)\FMRIB’s Diffusion Toolbox (FDT toolbox). Images were first corrected for motion and eddy current distortions. Diffusion parameters were calculated at each voxel, accounting for crossing fibers in two directions, using BEDPOSTX (Bayesian Estimation of Diffusion Parameters Obtained using Sampling Techniques) [32]. Separate probabilistic tractography analyses were performed between Lobule V and the contralateral motor subregion of the thalamus and then from the motor subregion of the thalamus to the ipsilateral PMC. A similar two-step approach was taken from Crus I/II to the contralateral thalamus and then on to the MFG. All tractography analyses were implemented in FSL using PROBTRACKX (Probabilistic tracking with crossing fibers) with a step length of 0.5 mm, 5000 streamlines from each voxel, a fiber threshold of 0.1, and modified Euler streamlining. Tracking was stopped when the streamline reached the edge of the brain mask, when tracking reached 2000 steps, or when the pathway exceeded ±80 degrees from one step [33,34].

All tractography was performed in individual subject space and then warped to standard MNI space using nonlinear transformations between individual subject diffusion space and MNI standard space. Individual masks were summed and divided by the total number of subjects to create the group probability map. This procedure was performed for each tract. These maps were thresholded so that the tract passed through a given voxel in at least 50% of subjects. They were then visualized using FSL View [33]. Notably, these tracts were used as two segments. The first segment was from the cerebellum to the relevant region of the thalamus, and the second segment was from the thalamus to the cortex. Mean FA was extracted from each tract segment for all subjects. Fractional anisotropy (FA) is used in DTI studies and varies from 0 to 1. It describes the degree of anisotropy of diffusion. A value of 0 describes the isotropic movement of water molecules, which is unrestricted (or equally restricted) in all directions. A value of 1 means that diffusion is extremely anisotropic and is restricted in only one direction. It reflects the tendency for water to diffuse in one direction as opposed to randomly. The FA values are unitless because they are a ratio of diffusion coefficients.

### 2.4. Neuropsychological Evaluation

A trained technician supervised by an experienced neuropsychologist administered the NIH Toolbox cognitive and motor assessment. To investigate motor speed, participants complete the 9 Hole Pegboard Dexterity Test. To investigate processing speed, participants completed the Pattern Comparison Processing Speed Test from the NIH Toolbox [35]. To examine executive functioning, participants completed the List Sorting Working Memory Test from the NIH Toolbox, a working memory test with auditory and visual stimuli. Participants also completed the Flanker Inhibitory Control and Attention Test and the Dimensional Change Card Sort (DCCS) Test from the NIH Toolbox, measures of inhibitory control and cognitive flexibility, respectively. To evaluate episodic memory, youth completed the Picture Sequence Memory Test. To assess language-based skills, patients completed the Picture Vocabulary Test and the Oral Reading Recognition Test, respectively assessing receptive vocabulary and reading decoding. The fluid and crystallized composite scores of the NIH Toolbox were generated from the subtests. Fluid abilities are thought to be more driven by biological processes, and the fluid composite includes the subtests assessing processing speed, executive functioning, and episodic memory. Although there is rapid improvement during childhood, it remains quite fixed in adulthood. Crystallized abilities are thought to be more experience-dependent and gauge learned skills such as vocabulary and reading. In contrast to their fluid abilities, they continue to improve to some degree with age and experience [36]. 

### 2.5. Statistical Analysis

All statistical analyses were performed using the GraphPad Prism software. Of note, the cognition of the CHD cohort with respect to the control cohort was also analyzed using univariate *t*-test analysis of composite, age-corrected scores from the NIH Toolbox. 

The primary analysis consisted of analyzing the FA as well as the volume and diffusion parameters (MD, AD, and RD) of 4 defined cognitive tracts (left CrusI/II region of the cerebellum to right thalamus; right thalamus to right prefrontal cortex; right CrusI/II region cerebellum to left thalamus and left thalamus to left prefrontal cortex) and 4 motor tracts (left motor region of the cerebellum to right thalamus; right thalamus to right primary motor cortex; right motor region of the cerebellum to left thalamus and left thalamus to left primary motor cortex) and investigating differences between the same tract in the CHD group with respect to healthy controls. Multivariate linear regression analysis was used to assess differences in each tract segment with age as an independent variable. The b1 association coefficient is denoted as “Estimate” in the tables, and here we will set forth an association between the FA value of the target tract as the dependent variable and the CHD status as the independent variable, controlling for age. A positive coefficient demonstrates that the FA of the tract increases CHD status. A negative coefficient demonstrates an inverse relationship. (See Supplemental Methods for more details.)

The secondary analysis aimed to correlate FA in the cognitive tracts with cognitive outcome measures using the NIH Toolbox (composite scores and executive function measures). This correlation was investigated using a multivariate linear regression analysis and a post-hoc analysis of our imaging results, namely FA variations in the different tracts with the NIHTB score and elected subtest scores controlling for age and CHD status. Here, the Estimate will denote the correlation between the NIH Toolbox itemized test and the target cognitive tract. 

Our secondary aim also characterized age-related differences in FA values of cognitive and motor tracts within the entire cohort. To facilitate this analysis, the cohort was separated into three age groups: a school-age child group (age 6–10: n = 19 controls and 10 CHD patients), an adolescent group (age 11–15: n = 32 controls and 16 CHD patients), and a young adult group (age 16 and up: n = 13 controls and 19 CHD patients). Multivariate linear regression with age as an independent factor was used to compare the FA values of tracts as well as diffusion parameters within each age group between CHD and controls, adjusting for age and sex. In this analysis, the Estimate will denote the estimated regression coefficient that quantifies the association between the dependent variable, namely, the FA of the target tracts, and the independent variable, namely, the CHD status, while controlling for age differences within the target group. 

Significant differences will be denoted with asterisks. A *p* value < 0.05 is denoted with a *. 

## 3. Results

### 3.1. Clinical and Neurocognitive Characterization of the Cohort

The final cohort analysis included 53 youths with CHD and 73 healthy peers. A total of thirty-two patients in the CHD group had cyanotic lesions (twelve Hypoplastic Left Heart Syndrome (HLHS), ten Transposition of the Great Arteries (d-TGA), three Tetralogy of Fallot (ToF), one truncus Arteriosus, five Double outlet right ventricle, and one tricuspid atresia), and twenty-one had a cyanotic lesion (eleven Ventricular Septal Defect (VSD), seven Coarctation of the Aorta, and three Atrial Septal Defect (ASD)) (Scheme S1). There were no differences in socio-demographic characteristics, including annual income and level of maternal education, between CHD and controls. However, 100% of all participants in the CHD cohort were white, while in the control cohort, 38% identified as non-white (Black or African American, Biracial, Asian, Hispanic, or Latino). 

NIH Toolbox assessment for the whole cohort using age-corrected crystallized, fluid, and total composite scores demonstrated a significant decrease in general neurocognitive tests of CHD patients (105.6 vs. 114.9 *p =* 0.0003; 97.88 vs. 106.7 *p =* 0.0199; and 101.7 vs. 113.7 *p =* 0.0018) with respect to the control cohort, respectively (See Table 1). When looking at the NIH Toolbox Total Cognition composite score corrected for age in the three age groups, CHD patients performed significantly worse than controls in the adolescent age group and the young adult age group (*p* < 0.05 in both cases) (Scheme S3). In the adolescent group, poorer episodic memory (*p* < 0.05) and language (*p* < 0.05) on select cognitive and behavioral measures were denoted. In the young adult group, slower executive function measured by DCSS (*p* < 0.01) and poorer episodic memory and language abilities (*p* < 0.05) were measured. Interestingly, both fluid and crystalized scores were also significantly lower in the young adult population (*p* < 0.05) and represent both lower biological and experience-driven abilities in cohorts with comparable socio-economic status. When looking at the motor subtest, there were no significant differences noted.

### 3.2. Cognitive and Motor Tract Imaging Data

#### 3.2.1. FA Measures in the Cognitive Loop: White Matter Tracts

**Figure 2:**
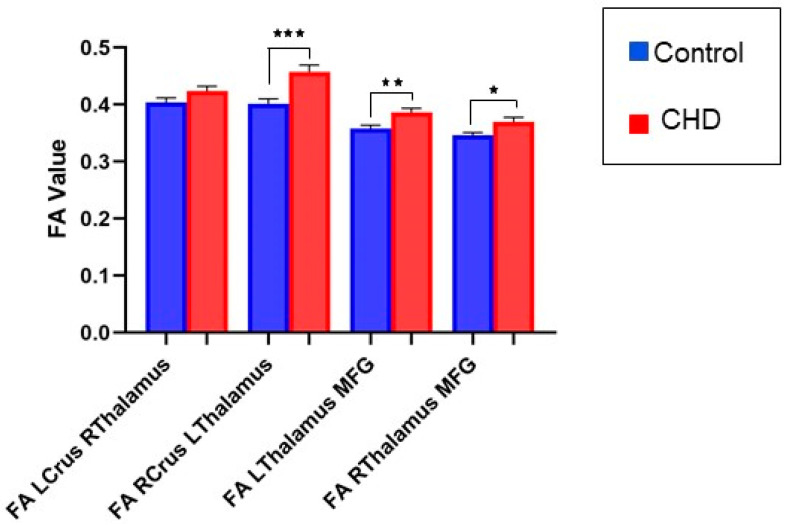
FA value in the cognitive loop. Data demonstrate a higher FA in the CHD population on all tracts of the cognitive loop with respect to the control group. “Cognitive tracts” are tracts emerging from left CrusI/II region of the cerebellum (LCrus) to the contralateral right thalamus (Rthalamus); right thalamus (Rthalamus) to middle frontal gyrus (MFG); right CrusI/II region of the cerebellum (RCrus) to the contralateral left thalamus (Lthalamus); and left thalamus (Lthalamus) to middle frontal gyrus (MFG). CHD cohort is denoted in red and control cohort in blue. * *p* < 0.05, ** *p* < 0.01, *** *p* < 0.001.

We noted a significant increase in FA in the white matter tract from the right cerebellum to the left thalamus in the CHD group compared with controls (Right Crus I/II to Left Thalamus) (*p* = 0.0005) (Figure 2). We also noted a significant increase in FA in the white matter tract from the left thalamus to the left prefrontal cortex (medial frontal gyrus—MFG) (*p* = 0.0086). There was no difference in FA in the white matter tract from the left cerebellum to the right thalamus in the CHD group compared with the control group (contralateral left Crus I/II to right thalamus) (*p* = 0.11) (Figure 2). In contrast, there was a slight increase in FA in the white matter tract from the right thalamus to the right prefrontal cortex (medial frontal gyrus-MFG) (*p* = 0.02642) (Figure 2) (Table 2). Diffusion parameters and Volume in the native and MNI spaces of tracts did not depict any significant differences (Supplementary Tables S1 and S2).

#### 3.2.2. FA Measures in the Motor Loop: White Matter Tracts

**Figure 3:**
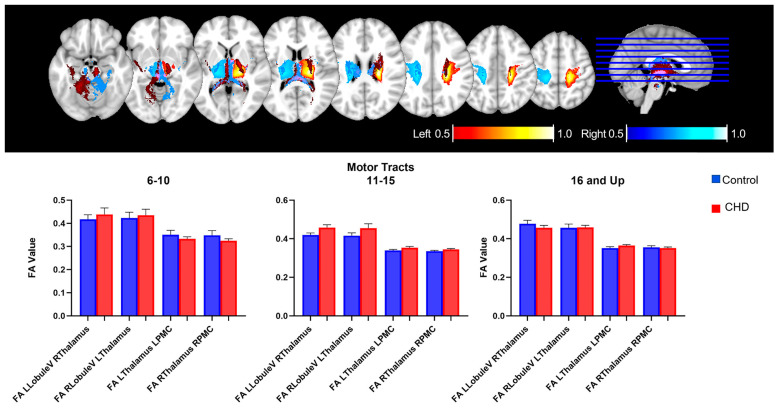
Probability Map of tracts seeding from the Lobule V region of the cerebellum to the contralateral primary motor cortex. There are no significant differences in the motor loop in any age group. “Motor tracts” are denoted as follows: left motor region of the cerebellum (L Lobule V) to right thalamus (R Thalamus); right thalamus (R thalamus) to right primary motor cortex (R PMC); right motor region of the cerebellum (R Lobule V) to left thalamus (L Thalamus); and left thalamus (L Thalamus) to left primary motor cortex (L PMC). A pediatric group (age 6–10, n = 19 controls and 10 CHD patients), an adolescent group (age 11–15, n = 32 controls and 16 CHD patients), and a young adult group (age 16 and up, n = 13 controls and 19 CHD patients).

We measured the total FA value in bilateral motor tracts emerging from Lobule VII/VIII of the cerebellum to the contralateral thalamus and then from the bilateral thalamus to the bilateral ipsilateral primary motor cortex (PMC) region (Figure 3) (Table 2). Previous probabilistic tractography works have used this tract as a reference within a control population because it is consistently reproducible. There were no significant differences in the FA value of the bilateral motor loop white matter tracts between the CHD group and controls in the cohort as a whole or when separated into three age groups, as detailed below. Interestingly, we did note significant decreases in volume in bilateral motor loop tracts from the thalamus to the right PMC in the CHD group compared with controls (Supplementary Table S1). With respect to diffusivity, we see a decrease in radial diffusivity as well as a decrease in axial diffusivity in the CHD group compared with controls (Supplementary Table S2).

### 3.3. Neurocognitive Assessment and Cerebello-Cerebral Cognitive Loop

The increase in FA from the right thalamus to the MFG tract in the cognitive loop (posterior subdivision) seen in the CHD group compared with controls significantly correlated with lower scores on the NIHTB total composite score test (*p* = 0.002), fluid score test (*p* = 0.0067), crystallized score test, and flanker inhibitory test (*p* = 0.0117) (Table 3). 

### 3.4. Age-Related Neuro-Cognitive and FA Measures in the Cognitive Loop

**Figure 4:**
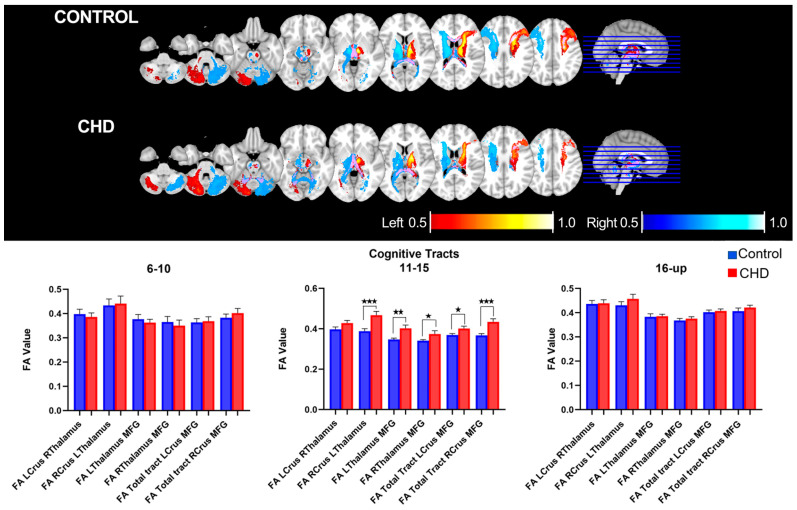
Probability Map of tract seeding from the CrusI/II region of the cerebellum to the MFG and FA value of cognitive loop tracts according to age group: A pediatric group (age 6–10 n = 19 controls and 10 CHD patients), an adolescent group (age 11–15 n = 32 controls and 16 CHD patients), and a young adult group (age 16 and up n = 13 controls and 19 CHD patients). There are no significant differences in FA value of tracts in both the pediatric and young adult populations. However, significantly higher FA values were noted specifically in the adolescent group in tracts of the cognitive loop of the CHD population vs. the control. “Cognitive tracts” are tracts emerging from left CrusI/II region of the cerebellum (LCrus) to right thalamus (Rthalamus); right thalamus (Rthalamus) to middle frontal gyrus (MFG); right CrusI/II region of the cerebellum (RCrus) to left thalamus (Lthalamus); and left thalamus (Lthalamus) to middle frontal gyrus (MFG). Lastly, total tracts represent the sum and average FA of the two half tracts from the Crus I/II region to the MFG. CHD cohort is denoted in red and control cohort in blue. * *p* < 0.05, ** *p* < 0.01, *** *p* < 0.001.

The cohort was then separated into three groups according to age: 6–10, 11–15, and 16 and up. The child (6–10) group was composed of 19 controls and 10 CHD patients; the adolescent (11–15) group was composed of 32 controls and 16 CHD patients. Finally, the young adult (16 and up) group was composed of 13 controls and 19 CHD patients. There are no significant differences in the FA value of tracts in both the child and young adult groups (Table 4). However, significant differences were noted specifically in the adolescent group, with an average higher FA in tracts of the cognitive loop of the CHD population vs. the control (Figure 4). 

## 4. Discussion

Children and adolescents with CHD often display cognitive and motor skill impairments, such as executive function deficits and behavioral and adaptive modifications. In our study, we tentatively investigated whether alterations in connectivity between the prefrontal and cerebellar brain structures could exist between CHD and control cohorts and if these alterations could predict cognitive outcome (cognitive loop) or motor outcome (motor loop) among youths with CHD. In the CHD group, fractional anisotropy (FA) was increased in the cognitive loop connectivity pathways compared with the healthy control group. In contrast, there were no differences between CHD and controls in motor loop connectivity pathways. An increase in FA in the cognitive loop (posterior subdivision) predicted lower scores on the NIHTB tests, including those of executive function. A transient increase in connectivity of the cognitive loop in the adolescent group was observed relative to the child and adult groups. While this age group analysis was exploratory and lacking in power for a definitive answer, our results suggest that selective alteration of cerebellum-cerebral connectivity circuitry within the cognitive loops predicts cognitive dysfunction in CHD youths. At this time, other factors could come into play, such as demographic factors and the type of intervention and lesion. Nevertheless, these findings may suggest a critical period of cerebellar circuitry plasticity in the adolescent period in CHD subjects that drives neurocognitive function. Future work with an increased sample size will allow us to conclusively demonstrate this. Of note, we also used an automated probabilistic tractography pipeline, which has been shown to provide more accurate tractography measures compared with standard deterministic tractography pipelines. 

### 4.1. Adolescence: A Critical and Exclusive Period

Our initial results demonstrated a cohort-wide increase in FA in CHD patients on the right cerebello-thalamic tracts as well as the thalamic-to-cerebral tracts. Interestingly, when we separated our CHD cohort into three subgroups by age, a significant increase in FA was visible specifically in the adolescent population. While previous studies using DTI and VBA demonstrated microstructural brain dysmaturation in infants with CHD correlated with decreased FA [37,38,39], the increase in FA we have demonstrated in the adolescent CHD population is in line with a previous study that showed hyperconnectivity and therefore increased FA within the region of the right inferior occipito-frontal fasciculus (IFOF) and right inferior longitudinal (occipito-temporal) fasciculus (ILF) of later preterm versus term infants [40]. One hypothesis to explain these results might be the ineffective pruning of synapses naturally occurring in the adolescent brain [39]. This pruning of unused or inefficient synapses is responsible for the maturation and efficient proficiency of the nervous system [41] and results in less tortuous fibers. In autism, increased dendritic spine density has been inversely correlated with cognitive function [42]. This increased spine density could be due to a lack of pruning leading to an immature network and therefore excessive excitatory connectivity [43]. Moreover, another study investigating prenatal injury through valproic acid exposure demonstrated hyperconnectivity and a hyperexcitable network [44]. Therefore, the absence of normal synaptic pruning may lead to neural hyperactivity and hyperconnectivity, leading to neurocognitive deficits. Importantly, while our results may hint at a critical period during adolescence, our small sample size may not allow us to conduct a more thorough analysis at the moment. Indeed, while the transient increase during adolescence may suggest a critical period, many other factors may be at play. Future work will strive to investigate this potential transient increase by increasing our sample size.

Lastly, we have demonstrated an increase in FA in the CHD population. While FA is widely used as a measure of connectivity, other factors may contribute to higher FA, such as myelination. Myelination is a widely known proxy for connectivity, axonal integrity, and signal-strength transmission. Increased FA is often associated with increased white matter myelination. However, increased myelination is also associated with increased RD, which we do not see in this study. Other factors may also induce an increased FA, such as decreases in axonal diameter, packing density, and branching. A future investigation of those parameters could contribute to demonstrating the hypothesis that Increased FA is not always associated with better connectivity. 

### 4.2. Increase in Connectivity Inversely Correlate the Decrease in NIHTB Performance: The Relevance of Networks in Cognitive Outcome

In our study, the CHD patient cohort demonstrated lower NIH toolbox performance than controls in working memory, language, and executive function in socially matched cases but not in motor subtests. Inversely, we demonstrated an increase in connectivity of the cognitive loop correlating with the decrease in NIHTB performance. The most sensitive tract to these measures seemed to be the right thalamo-frontal tract, and the correlation was specific to the total composite score test, fluid score test, crystallized score test, and flanker inhibitory test. These results raised the question of the influence of a network on a specific outcome and its capacity to be modulated, namely by circuit plasticity. Indeed, these increases in FA might highlight an increase in the plasticity of these networks to compensate for or modulate long-term cognitive outcomes that would otherwise be damaged. Interestingly, a recent review has set forth the idea of a functionally integrated network composed of the basal ganglia, the cerebellum, and the cerebral cortex. The network can be visualized as motor, affective, and cognitive nodes interconnected together. This network being a highly plastic structure, one may think of cognitive outcomes as the sum of multiple inputs coming from different brain regions. As such, if one region is damaged, to maintain or modulate the cognitive outcome, another region might increase or decrease its connectivity to compensate for the lack of input from this damaged region. In contrast, abnormal activity at one node can have network-wide effects [45]. This circuit plasticity theory can be applied in two ways in our study. On the one hand, the hyperconnectivity of the cerebello-cerebral tracts can be the error that needs to be compensated by other circuit mechanisms, or it can be the compensation for a deficiency in the network elsewhere. Taken together, these results do suggest that the presence of CHD does differentially impact specific sensorimotor unimodal circuitry versus hetero-modal or multi-modal regions via cortical and subcortical pathways, as seen here in relation to the cerebellum. To that end, a future aim will be to mainly look at the correlation between the tracts and these deficits in age-specific groups; another aim would be to look at other aspects of connectivity to have a broader and more complete approach using fMRI. Evaluation of the resting state connectivity in CHD patients vs. controls would contribute to a functional evaluation of the connectivity after our anatomical study. 

### 4.3. Cognitive Performance in the Adolescent and Young Adult Population

In this study, patients with CHD demonstrated lower cognitive performance in the adolescent and young adult population, mainly in language, episodic memory, and executive function, linked to altered cerebello-cerebral connectivity. Interestingly, these three cognitive domains have been linked to specific regions of the caudate, lateral, and dorsolateral prefrontal cortex [46]. A future study of the different network connections in these regions would be highly beneficial to understanding cognition processes in the adolescent and young adult brain. Here, no significant difference in cognitive measures was noted in the pediatric population. Because development is not complete, it is possible that the consequences of in utero or fetal injury such as CHD only become significant in older children, such as late-maturing executive functioning skills [8] and behavior regulation abilities [47]. A significant decrease in both the fluid and crystallized composite scores was also noted. All composite scores have been age corrected because this may lead to unreliable results in studies that compare multiple age ranges. While the biologically driven fluid composite score differences can be solely attributed to brain dysmaturation and CHD, it is important to consider that the alteration in the crystallized score, which reflects educational and social experience, may be due to the experience of living outside of the normal, regular frame of a young adult. 

## 5. Limitations

The main limitation of this study is its small sample size. As such, we had limited power to detect subtle effects. Our control group, although socially and demographically matched, was small with regards to sample size. However, the motor loop FA values in our controls are within the range of previously published work [33,34]. Indeed, when looking at our individual age groups, our sample size decreased drastically. A future endeavor will be to recruit more candidates to strengthen our results in the three age groups we designed, namely the child, adolescent, and young adult populations. Another limitation is the variety of heart lesions. Although the major types of lesions are represented in an equal manner for all age groups, it would be interesting to look at connectivity and neurocognitive outcome with respect to not only age but also type of heart lesion.

## Figures and Tables

**Table 1:** Demographic information on the cohort of children and adolescents with CHD and normal controls.

	Controls	CHD	*p*
*n*	73	53	
Age	13.23	14.88	0.06726
Maternal Education	5.6	4.4	0.7523
Mean Household Income	3.644	3.04	0.10118
Total Cognition Composite	113.7	101.7	0.0023 *
Crystallized Cognition Composite	114.9	105.6	0.0004 *
Fluid Cognition Composite	106.7	97.88	0.0025 *
Dimensional Card Change Sort (DCCS)	102.1	96.45	0.0478 *
Flanker Inhibitory Control and Attention	100.2	98.79	0.5506
List Sorting	106.1	102.3	0.0999
Picture Sequence Memory	107.5	98.02	0.0028 *
Oral Reading	117	106	0.0003
Picture Vocabulary	107.8	103	0.0221 *
Pattern Comparison	99.35	97.95	0.7206
9-Hole Pegboard Dexterity	106.9	104.8	0.4128

Maternal education is coded according to the following rubric: 1 = less than HS diploma, 2 = HS diploma, 3 = some college, 4 = completed trade/vocational training, 5 = Associate’s degree, 6 = Bachelor’s degree, 7 = Master’s degree, 8 = Doctorate/professional degree. Household income is coded as follows: 0 ≤ $25,000 or below; 1 = $25,000 − $34,999; 2 = $35,000 − $49,999; 3 = $50,000 − $74,999; 4 = $75,000 − $99,999; 5 = $100,000 − $149,999; 6 ≥ $150,000. Numbers with asterisks indicate a significant difference in the value of *p* < 0.05.

**Table 2:** Cognitive and motor loop tract FA variation according to CHD status analysis for motor and cognitive pathways.

	Estimate	Standard Error	95% CI	*p* Value	R^2^
Cognitive Tracts					
LCrus_RThalamus	0.01471	0.01273	−0.01053 to 0.03995	0.2503	0.05678
RCrus_LThalamus	0.05415	0.01509	0.02422 to 0.08409	0.0005 *	0.1252
LThalamus_MFG	0.02672	0.009964	0.006952 to 0.04648	0.0086 *	0.1747
RThalamus_MFG	0.02113	0.009381	0.002522 to 0.03974	0.0264 *	0.1702
Motor Tracts					
LLobuleV_RThalamus	0.01867	0.01384	−0.008759 to 0.04610	0.1801	0.2675
RLobuleV_LThalamus	0.02038	0.01644	−0.01226 to 0.05301	0.2182	0.3336
LThalamus_LPMC	0.006271	0.007993	−0.009550 to 0.02209	0.4342	0.1442
RThalamus_RPMC	−0.002758	0.008069	−0.01873 to 0.01321	0.733	0.07514

Numbers with asterisk denote a significant difference in the value of *p* < 0.05. “Cognitive tracts” are tracts emerging from left CrusI/II region of the cerebellum (LCrus) to right thalamus (Rthalamus); right thalamus (Rthalamus) to middle frontal gyrus (MFG); right CrusI/II region of the cerebellum (RCrus) to left thalamus (Lthalamus); and left thalamus (Lthalamus) to middle frontal gyrus (MFG). “Motor tracts” are denoted as follows: left motor region of the cerebellum (L Lobule V) to right thalamus (R Thalamus); right thalamus (R thalamus) to right primary motor cortex (R PMC); right motor region of the cerebellum (R Lobule V) to left thalamus (L Thalamus); and left thalamus (L Thalamus) to left primary motor cortex (L PMC). “Estimate” in the table denotes an association coefficient between the FA value of the target tract as the dependent variable and the CHD status as the independent variable. Numbers with asterisks (*) indicate a significant difference in the value of *p* < 0.05.

**Table 3:** Correlation between NIHTB itemized test and FA value of cognitive tracts.

NIHTB Fluid Score	Estimate	Standard Error	95% CI	*p* Value	R^2^
LCrus_RThalamus	−8.756	31.86	−71.92 to 54.41	0.784	0.07693
RCrus_LThalamus	−3.288	24.41	−51.70 to 45.12	0.8931	0.09260
LThalamus_MFG	44.47	34.36	−23.61 to 112.6	0.1982	0.0765
RThalamus_MFG	98.57	35.69	27.84 to 169.3	0.0067 *	0.1186
NIHTB Crystallized Score					
LCrus_RThalamus	2.44	20.97	−39.14 to 44.02	0.9076	0.1390
RCrus_LThalamus	6.195	16.36	−26.25 to 38.64	0.7056	0.1594
LThalamus_MFG	24.64	22.55	−20.03 to 69.32	0.2767	0.1507
RThalamus_MFG	60.05	23.41	13.66 to 106.4	0.0117 *	0.1841
NIHTB Composite Score					
LCrus_RThalamus	−7.976	31.36	−70.17 to 54.22	0.7998	0.1368
RCrus_LThalamus	−0.5671	23.92	−48.02 to 46.88	0.9811	0.1618
LThalamus_MFG	45.54	33.7	−21.23 to 112.3	0.1793	0.1423
RThalamus_MFG	109.4	34.55	40.91 to 177.8	0.002 *	0.1932
NIHTB DCCS					
LCrus_RThalamus	−0.589	25.18	−50.51 to 49.33	0.9814	0.0617
RCrus_LThalamus	−0.5211	19.62	−39.42 to 38.38	0.9789	0.0540
LThalamus_MFG	34.89	28.41	−21.40 to 91.17	0.222	0.05001
RThalamus_MFG	53.37	29.49	−5.050 to 111.8	0.073	0.0562
NIHTB Flanker Inhibitory Test					
LCrus_RThalamus	−19.24	21.91	−62.69 to 24.21	0.382	0.0929
RCrus_LThalamus	−3.257	17.32	−37.60 to 31.09	0.8512	0.0610
LThalamus_MFG	44.21	23.93	−3.211 to 91.63	0.0674	0.3414
RThalamus_MFG	79.22	24.51	30.66 to 127.8	0.0016 *	0.0904

Numbers with asterisks indicate a significant difference in the value of *p* < 0.05. Estimate denotes the correlation between the FA value of the target tract and the NIH Toolbox itemized test while controlling for CHD status and age differences within the group.

**Table 4:** Correlation of FA values of cognitive tracts with CHD status according to age groups.

	Cognitive Tracts	Estimate	Standard Error	95% CI	*p* Value	R^2^
Age 6–10						
	LCrus- RThalamus	0.01867	0.01384	−0.008759 to 0.04610	0.9148	0.07885
	RCrus- LThalamus	0.03746	0.03626	−0.03775 to 0.1127	0.3129	0.05057
	LThalamus-MFG	0.003297	0.02032	−0.03885 to 0.04545	0.8726	0.07358
	RThalamus-MFG	0.01341	0.02664	−0.04184 to 0.06865	0.6198	0.1565
Age 11−15						
	LCrus- RThalamus	0.03114	0.0188	−0.006716 to 0.06900	0.1045	0.05812
	RCrus- LThalamus	0.07916	0.02133	0.03620 to 0.1221	0.0006	0.2349 *
	LThalamus-MFG	0.05516	0.01567	0.02359 to 0.08673	0.0010	0.2161 *
	RThalamus-MFG	0.03261	0.0128	0.006831 to 0.05838	0.0143	0.1532 *
Age 16 and Up						
	LCrus- RThalamus	0.0029	0.02286	−0.04385 to 0.04965	0.8999	0.06234
	RCrus- LThalamus	0.02731	0.02748	−0.02890 to 0.08352	0.3286	0.03437
	LThalamus-MFG	0.008608	0.01293	−0.01783 to 0.03505	0.5108	0.1528
	RThalamus-MFG	0.005372	0.01537	−0.02606 to 0.03680	0.01940	0.7292

“Cognitive tracts” are tracts emerging from left CrusI/II region of the cerebellum (LCrus) to right thalamus (Rthalamus); right thalamus (Rthalamus) to middle frontal gyrus (MFG); right CrusI/II region of the cerebellum (RCrus) to left thalamus (Lthalamus); and left thalamus (Lthalamus) to middle frontal gyrus (MFG). Numbers with asterisks indicate a significant difference in the value of *p* < 0.05. In this table, “Estimate” depicts the association coefficient between the FA value of the tract and the CHD status. Numbers with asterisks (*) indicate a significant difference in the value of *p* < 0.05.

## Data Availability

The data presented in this study are available on request from the corresponding author. The data are not publicly available due to patient privacy.

## Supplementary Materials

The following supporting information can be downloaded at: https://www.mdpi.com/article/10.3390/jcm12165264/s1. Scheme S1: Heart Lesion Flow Chart; Scheme S2: Probabilistic tractography algorithm and pipeline creation: Workflow diagram show-ing the order and the elements used for each part of the pipeline process. The MNI Mask Directo-ry consisted of the masks of the Crus I/II region of the Cerebellum, the Lobule V region of the cerebellum, the Thalamus and the Middle Frontal Gyrus of the prefrontal cortex (See Figure 1). The Master Directory had seed, waypoint, and exclusion masks for each of the tracts outlined in Table 1; Scheme S3: NIH Toolbox itemized tests according to age. Upon looking at individual task performance; although no significant differences were noted in the pediatric group, notable differences were noted in the adolescent and young adult group. In the first group, poorer episodic memory (*p* < 0.05) and language (*p* < 0.05) on select cognitive and behavioral measures were denoted. In the young adult group, slower executive function measured by DCSS (*p* < 0.01) and poorer episodic memory and language abilities (*p* < 0.05) were measured. Interestingly, both fluid and crystalized scores were also significantly lower in the young adult population (*p* < 0.05) and represent both lower biological and experience driven abilities in cohorts with comparable socio-economic status; Table S1. Tracts Diffusion parameters; Table S2: Tracts Volume Parameters.
