# Supplementary material for: Increased Cerebello-Prefrontal Connectivity Predicts Poor Executive Function in Congenital Heart Disease
Source: J Clin Med. 2023 Aug 12;12(16):5264. doi: 10.3390/jcm12165264 (PMC10455623; doi:10.3390/jcm12165264)
Supplement: Supplementary file 1 [file jcm-12-05264-s001.zip › jcm-2469248-supplementary.pdf]

Journal of
Clinical Medicine

MDPI

Supplementary Materials

Increased Cerebello-Prefrontal Connectivity Predicts Poor Executive Function in Congenital Heart Disease

Aurelia Sahel [1,2], Rafael Ceschin [1,2], Daryaneh Badaly [3], Madison Lewis [1,2], Vince K. Lee [1,2], Julia Wallace [1], Jacqueline Weinberg [4], Vanessa Schmithorst [1], Cecilia Lo [5] and Ashok Panigrahy [1,2,6,*]

[1] Department of Radiology, University of Pittsburgh, Pittsburgh, PA 15213, USA; aurel117@gmail.com (A.S.); rcc10@pitt.edu (R.C.); mtl47@pitt.edu (M.L.); vkl2@pitt.edu (V.K.L.); julia.wallace@chp.edu (J.W.); vanessa.schmithorst@chp.edu (V.S.)
[2] Department of Biomedical Informatics, University of Pittsburgh, Pittsburgh, PA 15206, USA
[3] Child Mind Institute, New York, NY 10022, USA; daryaneh.badaly@childmind.org
[4] Department of Cardiology, University of Pittsburgh, Pittsburgh, PA 15261, USA; jacqueline.weinberg@chp.edu
[5] Department of Developmental Biology, University of Pittsburgh, Pittsburgh, PA 15201, USA; cel36@upmc.edu
[6] Department of Pediatric Radiology, Children's Hospital of Pittsburgh of UPMC, 45th Street and 4401 Penn Avenue, Pittsburgh, PA 15224, USA
* Correspondence: panigrahya@upmc.edu; Tel.: +1-412-692-5510; Fax: +1-412-692-6929

Supplemental Methods:

Statistical Analysis (Table 4 and Table 5):

Table 3 : Explanation of estimate variable : corrélation between the NIH Toolbox itemized test as a dependent variable with the FA value of a specific tract as the independent variable while controlling for CHD status and age differences within the group (Covariates). The "Estimate" value here represents the change in value of the specific NIHTB score associated to this tract. The "Estimate" value is the amount by which dependent variable (Here NIHTB score) will change if the independent variable (Here FA of tract) changes by 1. And since, FA is from 0-1 the range of the Estimate covers a wider range of NIHTB scores. The formula is: NIHTB score = Intercept + CHD Status + Age at MRI + FA of the targeted tract.

Table 4: Explanation of estimate variable : Here the correlation is between FA value of a specific tract (dependent variable) with CHD status is the independent variable while controlling for age (covariate) within each group. The" Estimate" provided in the table is change in FA Value between CHD patients (assigned value of 1) and Control patients (Assigned value of 0). Estimate should be between 0 and 1. The formula is: FA of specific tract = Intercept + CHD status + AGE AT MRI

Supplemental Figure Legends

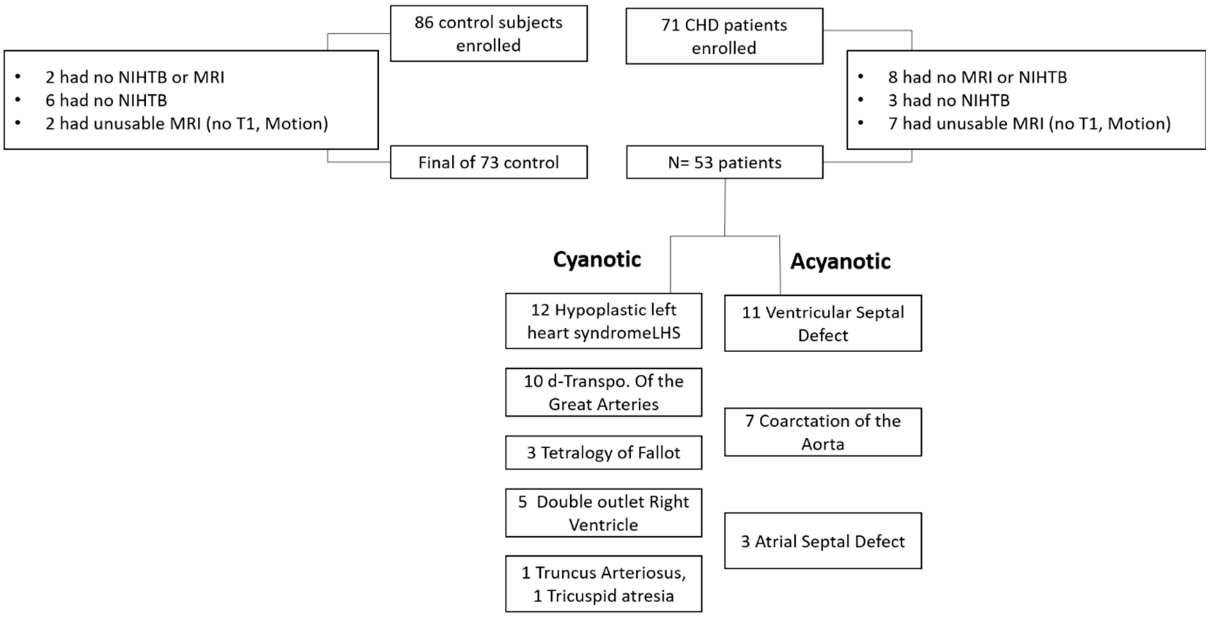

Scheme S1. Heart Lesion Flow Chart.

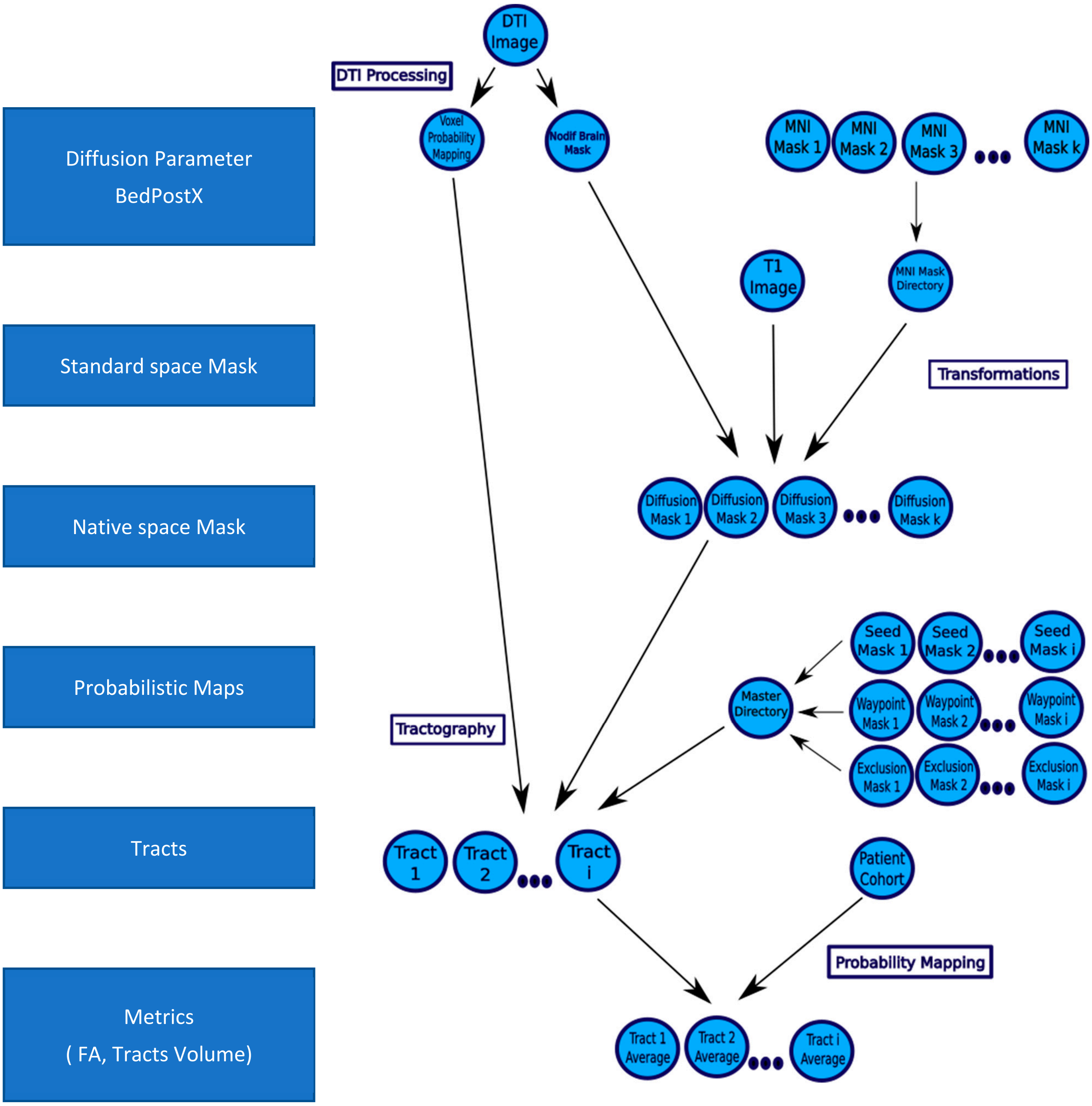

Scheme S2. Probabilistic tractography algorithm and pipeline creation: Workflow diagram showing the order and the elements used for each part of the pipeline process. The MNI Mask Directory consisted of the masks of the Crus I/II region of the Cerebellum, the Lobule V region of the cerebellum, the Thalamus and the Middle Frontal Gyrus of the prefrontal cortex (See Fig 1). The Master Directory had seed, waypoint, and exclusion masks for each of the tracts outlined in table 1.

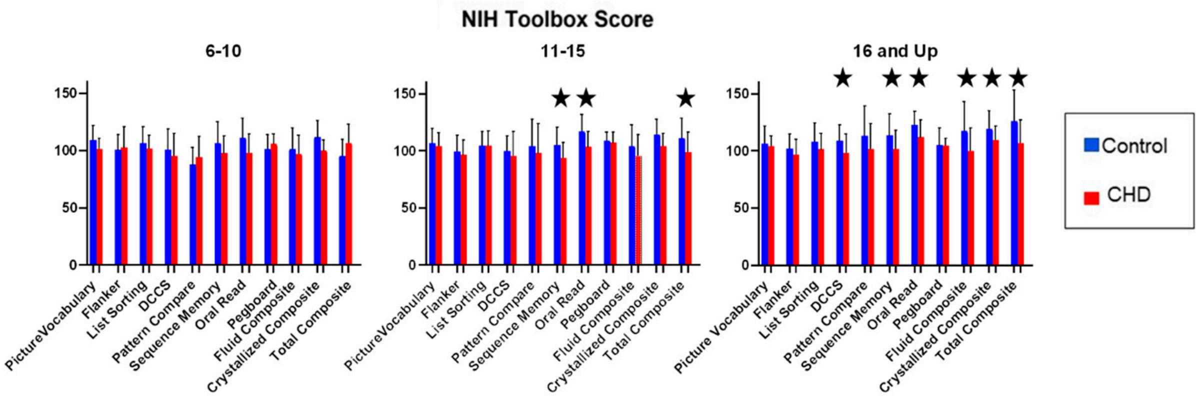

Scheme S3. NIH Toolbox itemized tests according to age. Upon looking at individual task performance; although no significant differences were noted in the pediatric group, notable differences were noted in the adolescent and young adult group. In the first group, poorer episodic memory ($p < 0.05$) and language ($p < 0.05$) on select cognitive and behavioral measures were denoted. In the young adult group, slower executive function measured by DCSS ($p< 0.01$) and poorer episodic memory and language abilities ($p< 0.05$) were measured. Interestingly, both fluid and crystalized scores were also significantly lower in the young adult population ($p < 0.05$) and represent both lower biological and experience driven abilities in cohorts with comparable socio-economic status.

Table S1. Tracts Diffusion parameters.

Tract	*LCrus_ RThalamus*	*RCrus_ LThalamus*	*LLobuleV_ RThalamus*	*RLobuleV _LThalamus*	*LThalamus_MFG*	*RThalamus_MFG*	*LThalamus_LPMC*	*RThalamus_RPMC*
MD CHD	8.86E-04	8.95E-04	9.48E-04	9.52E-04	8.64E-04	8.74E-04	9.17E-04	9.26E-04
StDev	9.30E-05	8.93E-05	1.21E-04	1.12E-04	6.62E-05	7.83E-05	8.37E-05	7.67E-05
MDControl	9.20E-04	9.22E-04	9.39E-04	9.51E-04	8.46E-04	8.48E-04	8.92E-04	8.94E-04
StDev	1.23E-04	1.15E-04	1.03E-04	1.24E-04	5.03E-05	4.91E-05	6.48E-05	6.18E-05
p	*1.06E-01*	*1.68E-01*	*6.89E-01*	*9.70E-01*	*8.75E-02*	*8.67E-02*	*6.87E-02*	*1.30E-02*
RD CHD	6.67E-04	6.39E-04	7.01E-04	7.02E-04	6.77E-04	6.99E-04	7.51E-04	7.65E-04
StDev	9.38E-05	1.07E-04	1.33E-04	1.18E-04	7.67E-05	8.96E-05	8.27E-05	7.91E-05
RD Control	7.03E-04	7.02E-04	7.06E-04	7.15E-04	6.74E-04	6.87E-04	7.34E-04	7.36E-04
StDev	1.17E-04	1.19E-04	1.11E-04	1.35E-04	6.18E-05	5.74E-05	7.13E-05	6.82E-05
p	*8.17E-02*	*2.49E-02* *	*8.23E-01*	*6.34E-01*	*8.05E-01*	*3.41E-01*	*2.21E-01*	*1.08E-01*
AD CHD	1.32E-03	1.36E-03	1.44E-03	1.45E-03	1.24E-03	1.23E-03	1.25E-03	1.25E-03
StDev	1.29E-04	1.30E-04	1.30E-04	1.35E-04	8.58E-05	8.89E-05	9.42E-05	7.93E-05
AD Control	1.36E-03	1.36E-03	1.41E-03	1.42E-03	1.19E-03	1.18E-03	1.21E-03	1.22E-03
StDev	1.76E-04	1.59E-04	1.49E-04	1.66E-04	8.25E-05	7.60E-05	7.34E-05	7.49E-05
p	*3.05E-01*	*9.46E-01*	*1.78E-01*	*3.62E-01*	*4.28E-03* *	*1.99E-03* *	*1.49E-02* *	*1.21E-02* *

Numbers with asterisks indicate a significant difference to the value of $p<0.05$.

Table S2: Tracts Volume Parameters.

	Tract Volume (Native space)					Tract Volume (MNI Space)				
Cognitive Tracts	CHD	St Dev	Controls	St Dev	*p*	CHD	St Dev	Controls	St Dev	*p*
LCrus_ RThalamus	5.01E+04	5.17E+04	3.95E+04	4.40E+04	*2.38E-01*	6.04E+04	5.95E+04	4.84E+04	5.00E+04	*2.47E-01*
RCrus_ LThalamus	4.26E+04	4.75E+04	3.55E+04	4.38E+04	*4.15E-01*	5.17E+04	5.80E+04	4.41E+04	5.45E+04	*4.85E-01*
LThalamus_ MFG	3.36E+04	2.72E+04	4.33E+04	2.75E+04	*5.48E-02*	4.38E+04	3.03E+04	5.47E+04	3.45E+04	*7.49E-02*
RThalamus_ MFG	3.60E+04	2.82E+04	4.33E+04	2.64E+04	*1.45E-01*	4.42E+04	3.21E+04	5.35E+04	3.23E+04	*1.18E-01*
MotorTracts										
LLobuleV_RThalamus	2.09E+04	2.69E+04	1.94E+04	2.62E+04	*7.84E-01*	2.54E+04	3.18E+04	2.39E+04	3.34E+04	*8.16E-01*
RLobuleV_LThalamus	1.81E+04	2.21E+04	1.76E+04	2.29E+04	*9.11E-01*	2.16E+04	2.64E+04	2.19E+04	3.04E+04	*9.50E-01*
LThalamus_ LPMC	2.79E+04	2.27E+04	4.15E+04	2.50E+04	*2.28E-03 **	3.52E+04	3.04E+04	5.19E+04	3.24E+04	*4.31E-03 **
RThalamus_ RPMC	3.35E+04	2.52E+04	4.53E+04	2.73E+04	*1.60E-02 **	4.01E+04	2.89E+04	5.63E+04	3.47E+04	*6.75E-03 **

Numbers with asterisks indicate a significant difference to the value of $p<0.05$.
